# New Bacteriocins from *Lacticaseibacillus paracasei* CNCM I-5369 Adsorbed on Alginate Nanoparticles Are Very Active against *Escherichia coli*

**DOI:** 10.3390/ijms21228654

**Published:** 2020-11-17

**Authors:** Yanath Belguesmia, Noura Hazime, Isabelle Kempf, Rabah Boukherroub, Djamel Drider

**Affiliations:** 1 UMR Transfrontalière BioEcoAgro1158, Univ. Lille, INRAE, Univ. Liège, UPJV, YNCREA, Univ. Artois, Univ. Littoral Côte d’Opale, ICV—Institut Charles Viollette, F-59000 Lille, France; yanath.belguesmia@univ-lille.fr (Y.B.); noura.hazime@hotmail.fr (N.H.); 2 Univ. Lille, CNRS, Centrale Lille, ISEN, Univ. Polytechnique Hauts-de-France, UMR 8520—IEMN, F-59000 Lille, France; rabah.boukherroub@univ-lille.fr; 3 ANSES, Laboratoire de Ploufragan-Plouzané-Niort, Unité Mycoplasmologie Bactériologie Antibiorésistance, 22440 Ploufragan, France; Isabelle.KEMPF@anses.fr

**Keywords:** new bacteriocins, alginate nanoparticles, nano-antibiotic, cytotoxicity, Gram-negative bacteria, antibacterial

## Abstract

*Lacticaseibacillus paracasei* CNCM I-5369, formerly *Lactobacillus paracasei* CNCM I-5369, produces bacteriocins that are remarkably active against Gram-negative bacteria, among which is the *Escherichia coli-*carrying *mcr*-1 gene that is involved in resistance to colistin. These bacteriocins present in the culture supernatant of the producing strain were extracted and semi-purified. The fraction containing these active bacteriocins was designated as E20. Further, E20 was loaded onto alginate nanoparticles (Alg NPs), leading to a highly active nano-antibiotics formulation named hereafter Alg NPs/E20. The amount of E20 adsorbed on the alginate nanoparticles was 12 wt.%, according to high-performance liquid chromatography (HPLC) analysis. The minimal inhibitory concentration (MIC) values obtained with E20 ranged from 250 to 2000 μg/mL, whilst those recorded for Alg NPs/E20 were comprised between 2 and 4 μg/mL, which allowed them to gain up to 500-fold in the anti-*E. coli* activity. The damages caused by E20 and/or Alg NPs/E20 on the cytology of the target bacteria were characterized by transmission electron microscopy (TEM) imaging and the quantification of intracellular proteins released following treatment of the target bacteria with these antimicrobials. Thus, loading these bacteriocins on Alg NPs appeared to improve their activity, and the resulting nano-antibiotics stand as a promising drug delivery system.

## 1. Introduction

Antibiotic resistance and its rapid dissemination worldwide have become a major societal threat that must be taken into consideration in order to not jeopardize the progress of modern medicine in the fight against the infectious diseases. Antibiotic resistance, which is occurring around the world, is undoubtedly associated with the massive and inconsiderate use of these drugs for many years and clearly endangering the efficacy of these molecules, which have transformed medicine and saved millions of lives [1].

According to a recent study published in 2019, in the European Union (EU) and European Economic Area (EEA), the infections attributable to antibiotic-resistant bacteria were responsible for more than 33,000 deaths in 2015, and this burden represents 170 disability-adjusted life-years (DALYs) per 100,000 population [2]. Bacteria with the highest effect on the health in 2015 were the third-generation cephalosporin-resistant *Escherichia coli*, methicillin-resistant *Staphylococcus aureus* (MRSA)*,* carbapenem-resistant *Pseudomonas aeruginosa*, and third-generation cephalosporin-resistant *Klebsiella pneumoniae* [2].

In the United States, another recent study outlined that 162,044 people die from multi-drug-resistant infections yearly in this country [3], which makes a huge difference regarding the 23,000 deaths occurring yearly and usually reported by the Center for Diseases Control and Prevention. Moreover, resistance to antibiotics has been observed for all classes of antibiotics [4], including those considered of last resort, such as colistin. Indeed, colistin was shelved from clinical practice in human medicine by the Food and Drug Administration (FDA) in 1959, because of its adverse effects. Colistin was then replaced by antibiotics belonging to the families of aminoglycosides, quinolones, and β-lactams, which were considered to be less cytotoxic and very active against Gram-negative bacilli (GNB). However, the emergence of multidrug-resistant GNB and the lack of new antibiotics to eradicate infections due to these microbes have led to the revival of polymyxins, an old class of cationic, cyclic polypeptide antibiotics [5]. Unlike human medicine, colistin has never been withdrawn from the veterinary circuit and has largely been used for the prevention and treatment of certain GNB infections, and even as a growth factor particularly in poultry and pig farming sectors [6,7]. Since 2015, a transferable system of colistin resistance has been discovered in *E. coli* strains, of human and animal origin, isolated in China [8]. This study has revealed that colistin resistance was attributed to the *mcr*-1 plasmid gene encoding the synthesis of a phosphoethanolamine transferase [8]. Shortly after the detection of the *mcr*-1 gene in China, new strains carrying this gene have been isolated everywhere around the world [9,10]. Although the genetic support of the *mcr*-1 gene is most often on plasmid DNA [11], its chromosomal carriage has also been reported in some strains [12]. Then, a plethora of *mcr* genes (*mcr*-2 to *mcr*-8) and their variants have been identified in *E. coli* strains, but also in other *Enterobacteriaceae* [13]. Very recently, the *mcr*-9 and *mcr*-10 genes have been reported, respectively, in a strain from human origin identified as *Salmonella enterica* serotype Typhimurium and in an *Enterobacter roggenkampii* clinical strain [14,15].

Since the discovery of the *mcr*-1 gene and its rapid spread throughout the world, several actions have been taken. For example, China has decided to ban the use of colistin as a growth factor since 2016, paving the way for other countries [16].

Thereafter, antibiotic resistance is standing as a mounting problem, underscoring the immediate need for accelerated and strengthened investments, innovations, and public health efforts to fight this resistance. Alternate approaches including phage therapy, probiotics, antibodies, vaccines, and antimicrobial peptides (AMPs) have shown promising results that suggest the role of these alternatives as preventive or adjunct therapies in the near future [17].

Bacteriocins are antimicrobial peptides ribosomally synthesized by both GNB and Gram-positive bacteria (GPB) [18]. Most bacteriocins produced by GPB are from lactic acid bacteria (LAB); thus, LAB-bacteriocins are most often active against bacteria closely related to the producing strain. Nevertheless, a few studies have outlined their activities against genetically distant bacteria [19,20]. In terms of applications, LAB-bacteriocins have been associated with the food sector as additives [21], but different studies underpinned their role as a toolbox to synergize or replace fading antibiotics [22,23,24,25].

Here, we demonstrate that bacteriocins produced by *Lacticaseibacillus paracasei* CNCM I-5369, referred as the E20 fraction, are active against a panel of *E. coli* strains, and this activity is significantly enhanced through E20 adsorption on alginate nanoparticles (Alg NPs). 

## 2. Results and Discussion

### 2.1. Peptide Mapping of the E20 Fraction

**Figure 1:**
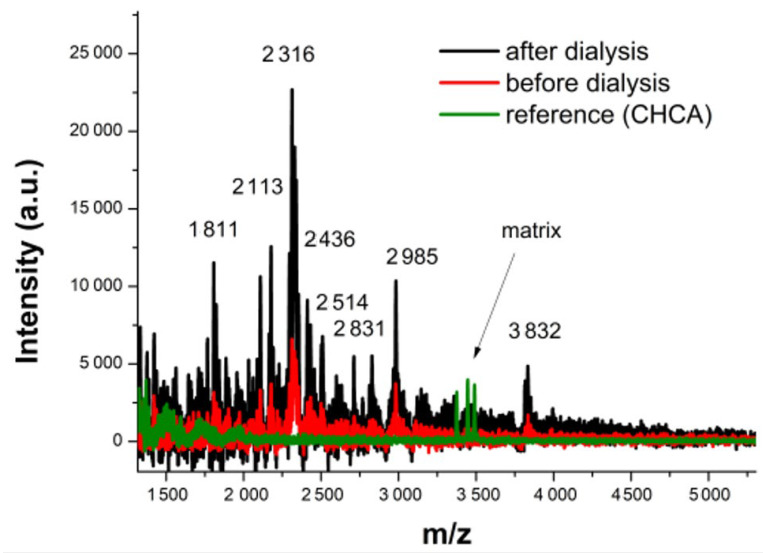
MALDI-TOF-MS (Matrix-Assisted Laser Desorption/Ionization-Time Of Flight-Mas Spectrometry) profile of E20 fraction before (red) and after (black) dialysis. A spectrum of the α-cyano-4-hydroxycinnamic acid (CHCA) matrix is also shown (green).

Mass spectrometry analysis of the E20 fraction detected several peptides with an m/z ratio of less than 4000, especially between 1811 and 2985, with an isolated peak at 3832 as seen in Figure 1. This analysis permitted us to detect at least 5 to 8 peptides of different sizes. However, this map analysis revealed no differences in peptide mapping before and after dialysis of the E20 fraction (Figure 1). Despite the sizes of these peptides, it remains nevertheless difficult to identify accurately the bacteriocins produced by *Lacticaseibacillus paracasei* CNCM I-5369. 

### 2.2. Characterization of Alginate Nanoparticles and Alginate Nanoparticles Loaded with E20

The encapsulation of LAB-bacteriocins has involved several methods to protect them from unfavorable environmental conditions and incompatibilities. The immobilization of the E20 fraction on Alg NPs does not have a considerable impact on their size. Indeed, the dynamic light scattering (DLS) measurement revealed an average size of alginate nanoparticles of 119 nm (pH 5) (Table 1), which increased to 124 nm after loading the E20 fraction. 

This slight increase of the nanoparticles’ size indicated E20 adsorption on the surface of the nanoparticles. These data were furthermore corroborated by the zeta potential measurements. The surfaces of the Alg NPs were negatively charged (−32 mV) at pH 7 and (−12 mV) at pH 5, and became neutral at pH 5 upon E20 adsorption on their surfaces. 

**Figure 2:**
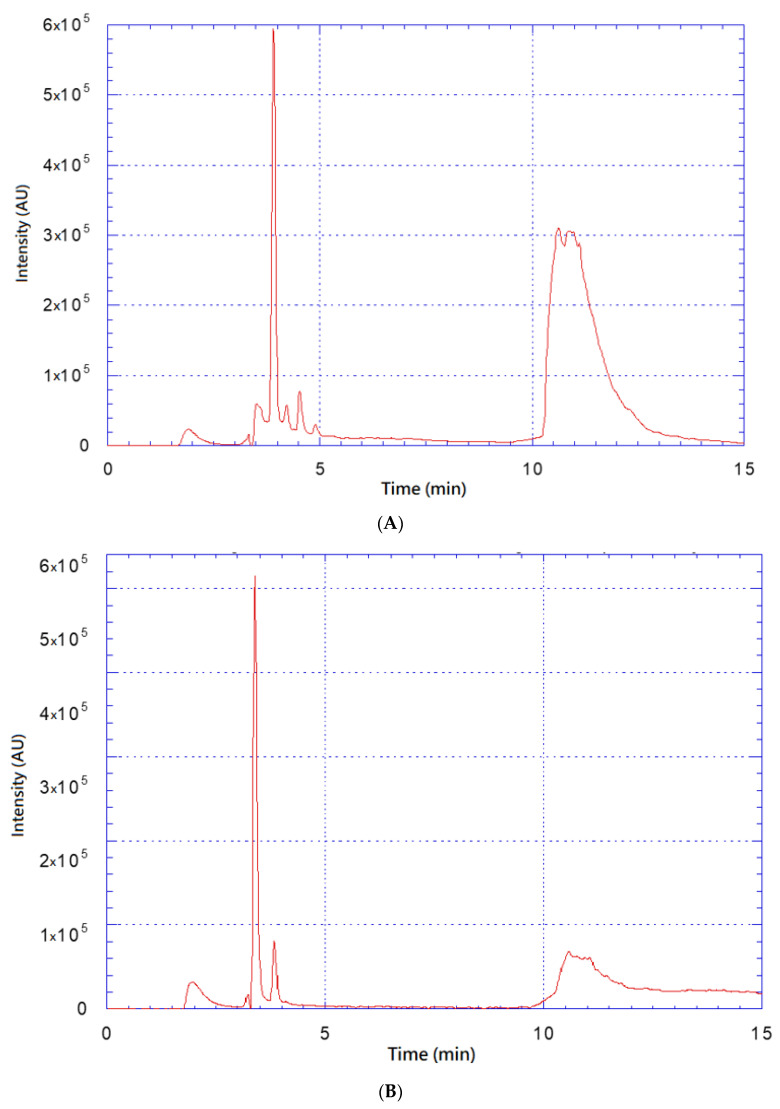
High-performance liquid chromatography (HPLC) chromatograms of the mixed E20 fraction and alginate nanoparticles (Alg NPs) before (**A**) and after dialysis (**B**) on 8 kDa cellulose membrane.

The high-performance liquid chromatography (HPLC) analysis of the mixed E20 fraction and Alg NPs was performed before and after dialysis on an 8 kDa cellulose membrane, in order to remove the non-adsorbed E20 compounds on Alg NPs. This analysis showed a first peak corresponding to the Alg NPs (at 4 min) before and after dialysis, as depicted in Figure 2A,B. Notably, before dialysis, a large peak corresponding to the E20 fraction was also detected and visualized after 10 min (Figure 2A). Interestingly, after dialysis the area of this peak was largely reduced (Figure 2B) due to the elimination of un-adsorbed E20 on Alg NPs, allowing us to determine that a maximum amount of 60–64 µg/mL of E20 was loaded on 500 µg/mL of Alg NPs. 

**Figure 3:**
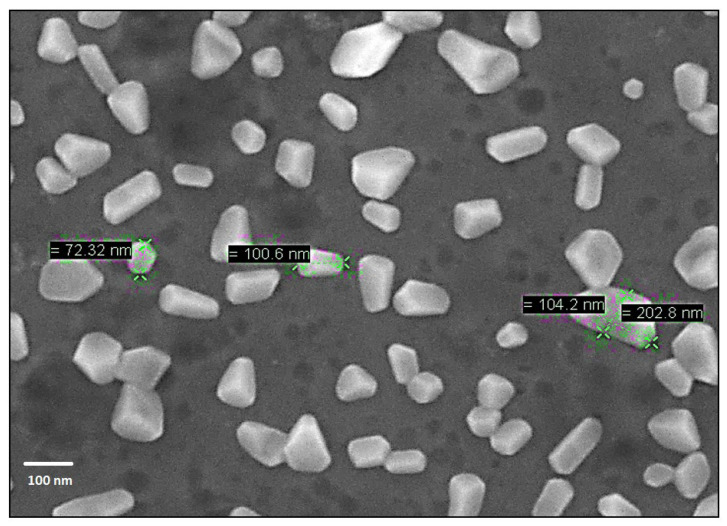
Scanning electron microscopy image of the alginate nanoparticles.

The size and morphology of the Alg NPs were also studied by scanning electron microscopy (SEM) imaging (Figure 3). The SEM image revealed various forms (rectangular and rod shaped) of the synthesized nanoparticles. Deeper analysis indicated that the average sizes of the rectangular and rod-shaped nanoparticles were 100 and 108 nm, respectively (data not shown). 

### 2.3. E20 Association with Alginate Nanoparticles’ Anti-E. coli Activity at Different pH Values 

LAB-bacteriocins are known for their activity over a wide range of pH values [26]. Nonetheless, antibacterial activity of E20 fraction was only visualized at pH 4.5–5. This pH-dependence was also reported for other LAB-bacteriocins such as plantaricin 163 and bacteriocin Si3 [27,28]. Overall, cationic LAB-bacteriocins were described as being active at acidic pH [29,30]. Assays were conducted using the following weight ratio of Alg NP/E20: 500/60 µg/mL. Afterwards, the antibacterial activity of Alg NPs/E20 was tested at different pH values against *E. coli* 184 as the target strain, using the well-known agar diffusion method (Table 2) [31]. The strong antibacterial activity was confirmed at pH 5, nevertheless, and interestingly, a slight activity was registered at other pH values.

### 2.4. Determination of Minimal Inhibitory Concentration (MIC) against a Panel of E. coli Strains and Development of a Nano-Antibiotic Formulation

To characterize the spectrum of antibacterial activity of E20, different *E. coli* strains have been used as target bacteria. This panel, as shown on Table 3, includes strains with different phenotypes. Encouragingly, the E20 fraction, produced by *Lacticaseibacillus paracasei* CNCM I-5369, exhibited activity against the aforementioned target bacteria with MIC values comprised between 250 and 2000 µg/mL when pH was adjusted at 5 (Table 3). It is noteworthy that LAB-bacteriocins were rather well-characterized for their antibacterial spectra directed against GPB. To improve the antibacterial activity registered against *E. coli* strains, the E20 fraction was loaded on alginate nanoparticles’ (Alg NPs) surface, leading to an Alg NPs/E20 nano-antibiotic formulation. In this layout, the antibacterial activity significantly increased based on the MIC values, which decreased by 500-fold (Table 3). The MIC values obtained with Alg NPs/E20 were comparable to, or even better than, those recorded with colistin alone (Table 3). Nevertheless, Alg NPs alone were devoid of any antibacterial activity (data not shown).

### 2.5. Antibacterial Activity 

**Figure 4:**
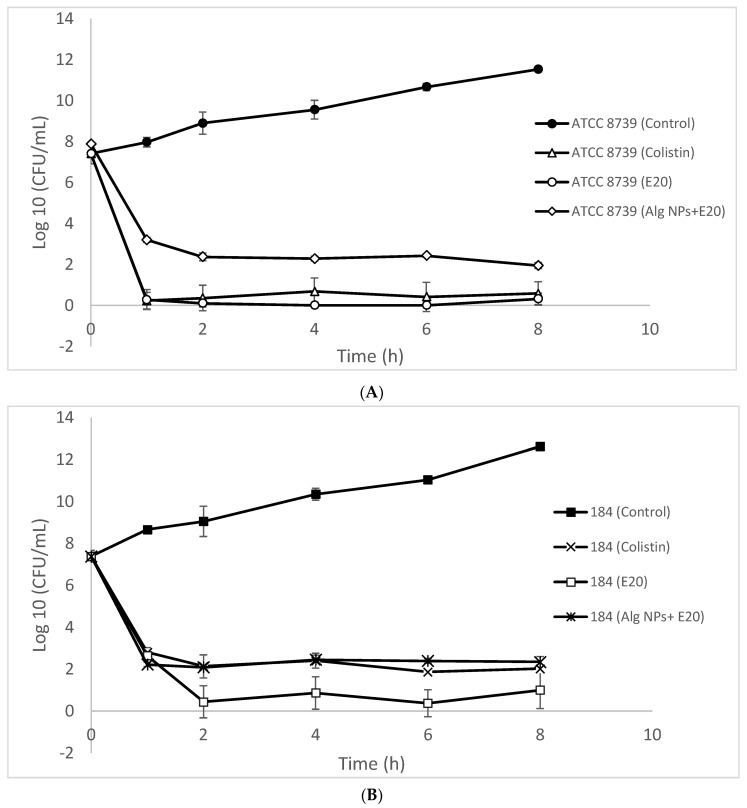
Time-killing curves established for *E. coli* ATCC 8739 (**A**) and *E. coli* 184 (*mcr-*1) (**B**) strains treated (white symbols) or not (full black symbols) with E20 fraction or colistin.

Antibacterial activity was studied through time-killing curves and minimum inhibitory concentration (MIC) of the E20 fraction alone and loaded on alginate nanoparticles (Alg NPs/E20). As indicated in Figure 4A, the number of CFU/mL rapidly decreased below 100 CFU/mL, reaching nearly 0 CFU/mL after 1 h of incubation, when the target bacteria, *E. coli* ATCC 8739, was treated either with the E20 fraction or colistin. This bactericidal effect was well observed for *E. coli* 184, which is a colistin-resistant strain, but after 2 h of incubation (Figure 4B). When these target bacterial strains were not treated with any of these antimicrobials, their growth occurred normally over time and reached 10^11^ to 10^12^ CFU/mL (Figure 4). 

The data gathered with Alg NPs/E20 prompted us to suggest this nano-antibiotic formulation as an innovative strategy to tackle infections caused by GNB, and more particularly *E. coli*. The EUCAST (European Committee on Antimicrobial Susceptibility Testing) clinical breakpoints and epidemiological cutoff of colistin were 2 µg/mL [32,33]. Therefore, strains with MIC values below or equal to this value were considered as sensitive to colistin, whereas those exhibiting MIC values greater than 2 µg/mL were considered as resistant to this antibiotic [34,35]. Alg NPs/E20 outlines once again the importance of LAB-bacteriocins as appropriate substances to consider against bacterial infections, as suggested in different reports [36,37,38,39,40,41]. The immobilization of the E20 fraction on alginate nanoparticles significantly enhanced their antibacterial activity. Additionally, oligosaccharides such as alginate biopolymer have been reported to be capable of impacting multi-resistant bacteria, rendering them more sensitive to the action of antimicrobial molecules [42]. 

### 2.6. E20 and E20 Adsorbed on Alginate Nanoparticles Modify E. coli Cell Morphology

**Figure 5:**
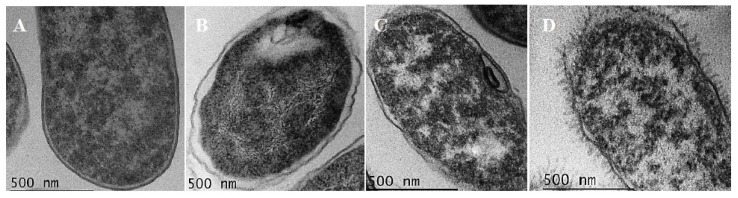
Transmission electron microscopy (TEM) images of untreated *E. coli* 184 cells (**A**) and those treated with (**B**) Alg NPs, (**C**) E20 fraction, and (**D**) nano-antibiotic Alg NPs/E20 formulation.

LAB-bacteriocins endowed with activity against *E. coli* strains, and more particularly against the *E. coli-*carrying *mcr*-1 gene have never been reported before. To gain insights on the E20 mode of action, we treated *E. coli* 184 with the E20 fraction alone, the Alg NPs/E20 nano-antibiotic formulation, and ultimately with Alg NPs as the control. Afterwards, cell morphology and ultrastructure were analyzed by transmission electron microscopy (TEM) imaging, as shown in Figure 5. In all treatments, morphological changes were registered, in comparison to the untreated sample. Related to that, non-treated *E. coli* 184 cells displayed typical bacterial cell form. Indeed, inner and outer membranes were intact, and the periplasmic space remained thin and uniform (Figure 5A). Nevertheless, after exposure of *E. coli* 184 to Alg NPs (Figure 5B), the inner and outer membranes remained intact, but the cytosol density increased. Cell damages started to be more pronounced, after exposure of bacterial cells to the E20 fraction at 1 mg/mL. Indeed, the inner and outer membranes still remained intact, but invagination of inner membrane started to be formed and the periplasmic space thickened comparatively to the control (Figure 5C). Ultimately, when the target bacteria were treated with Alg NPs/E20 (500/60 µg/mL), severe damages were registered such as lesions at the inner and outer membranes, and the periplasmic space was invaded by dense materials likely issued from the bacterial cytosol and leakage of intracellular material (Figure 5D).

**Figure 6:**
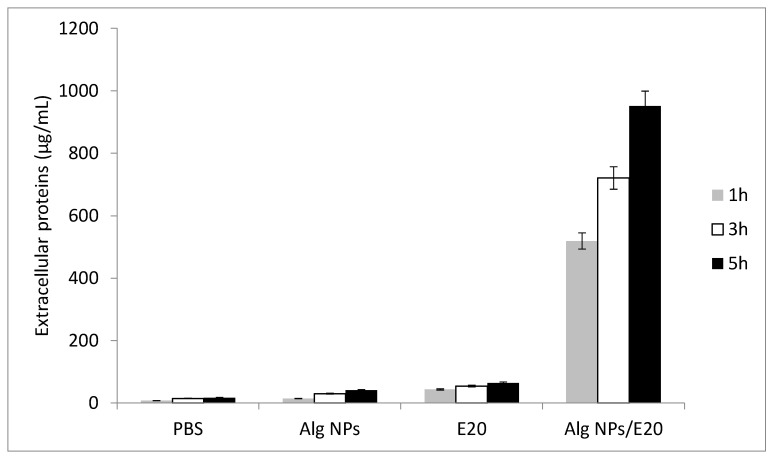
Amounts of extracellular protein concentration (µg/mL) after treatment of *E. coli* 184 with PBS, Alg NPs, E20 fraction, or Alg NPs/E20 nano-antibiotic formulation.

The second approach consisted of measuring the quantity of proteins released after treatment of *E. coli* 184 with the aforementioned antimicrobial substances. Notably, the control assay was conducted with PBS buffer at pH 5. After 1 h incubation, the concentrations of proteins were 7.49, 14.12, 43.53, and 518.92 µg/mL upon bacterial treatment with PBS, Alg NPs, the E20 fraction, and the Alg NPs/E20 nano-antibiotic formulation, respectively (Figure 6). After 3 h of exposure to these antimicrobials, the leakage was more pronounced with the E20 fraction and Alg NPs/E20. Indeed, the protein concentrations increased to 54.19 µg/mL for the E20 fraction and 720.72 µg/mL for Alg NPs/E20 (Figure 6). Ultimately, after 5 h of treatment, the concentrations of proteins were more important. In this case, the amounts of proteins were 64.01 and 951.36 µg/mL for samples treated with the E20 fraction and Alg NPs/E20, respectively, whereas the amount of proteins measured from samples treated with Alg NPs was 40.93 µg/mL. These analyses contribute to decrypt the mode of action of LAB-bacteriocins towards GNB. The combination of TEM analysis and protein quantification after treatment of *E. coli* 184 with the E20 fraction, Alg NPs, or the Alg NPs /E20 nano-antibiotic formulation prompted us to conclude that the antibacterial action occurred through intracellular protein leakage and cell membrane disruption. This mechanism was particularly important when the cells were treated with the Alg NPs/E20 nano-antibiotic formulation. Additional studies are nevertheless requested to fully understand this mechanism. 

### 2.7. Nano-Antibiotic Formulation Alg NPs/E20 Is Not Toxic for Human (HT29) and Animal (IPEC-1) Cell Lines

**Figure 7:**
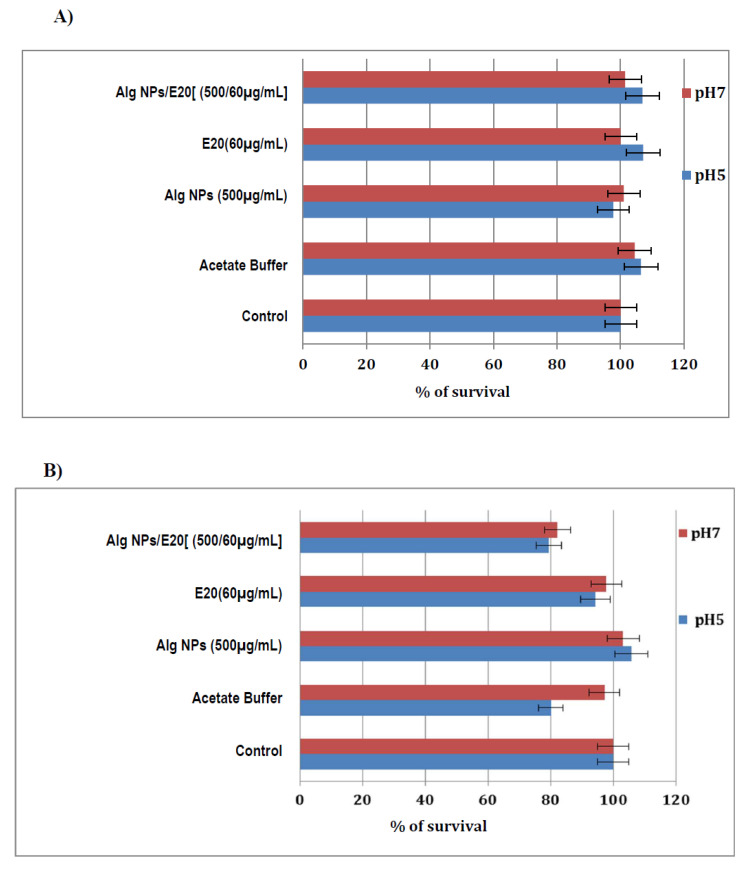
Survival percentage of human (HT29) (**A**) and animal (IPEC-1) (**B**) cell lines after treatment with acetate buffer, Alg NPs, E20 fraction, or Alg NPs/E20 at pH 5 and 7. Untreated control and treated control with acetate buffer were also tested at the same pH.

As indicated in Figure 7A, no deleterious effect was registered upon exposure of human HT29 cells to Alg NPs, the E20 fraction, or the Alg NPs/E20 nano-antibiotic formulation at pH 5 and 7. A cell viability of 100% was calculated from the positive control. At pH 5, this viability was nearly 98%, but this decrease did not seem to be statistically significant. On the other hand, IPEC-1 cells treated with Alg NPs, the E20 fraction, or the Alg NPs/E20 nano-antibiotic formulation exhibited different patterns (Figure 7B). A 100% of cell viability was registered for IPEC-1 cells treated with Alg NPs at pH 5 and 7. Then a slight and statistically insignificant decrease was observed when these cells were treated with E20 at 60 µg/mL. Ultimately, the viability of IPEC-1 cells decreased to 80%, when they were exposed to the Alg NPs/E20 nano-antibiotic formulation, at pH 7 and 5. This decrease in cell viability was not necessarily associated with the nature of the antimicrobial used, but rather with test conditions, as suggested by controls with acetate buffer. 

## 3. Materials and Methods 

### 3.1. Strains 

The bacteria used in this work are listed in Table 4. The bacteriocinogenic *Lacticaseibacillus paracasei* CNCM I-5369 was cultured in MRS (de Man, Rogosa and Sharpe) medium [43], without stirring, for 24 h at 37 °C. The *E. coli* strains, used as targets exhibiting different phenotypes for colistin resistance, were cultured in brain heart infusion (BHI) medium for 24 h at 37 °C.

### 3.2. Preparation of E20 Fraction and Its Analysis by Mass Spectrometry

The E20 fraction was obtained from the cell-free culture supernatant of the *Lacticaseibacillus paracasei* CNCM I-5369, grown as described previously, after centrifugation (8000 g, 4 °C, 10 min) and purification on a C18 reverse phase Bond Elut^TM^ cartridge (Agilent, Santa Clara, CA, USA). Briefly, 40 mL of the cell-free supernatant was loaded on the C18 reverse phase cartridge. The flow through was discarded and a first washing step was performed with 40 mL of 10% (*v*/*v*) of acetonitrile in ultrapure water in order to remove contaminants. Then the E20 fraction was eluted with 40 mL of 20% (*v*/*v*) of acetonitrile in ultrapure water, dried in SpeedVac under a vacuum with soft heating (40 °C), and then taken up in a volume of 4 mL of sterile distilled water. The E20 fraction was stored at 4 °C prior to use. The peptide content of the E20 fraction was analyzed by mass spectrometry before and after dialysis using an 8 kDa membrane. MALDI mass spectra were recorded on a Bruker Autoflex Speed instrument (Bruker Daltonics, Wissembourg, France) equipped with a TOF (Time of Flight) analyzer and LIFT module. A pulsed Smartbeam II 2 kHz laser at a wavelength of 355 nm (~3.49 eV) was operated at a frequency of 1000 Hz (MS data) with a delayed extraction time of 130 ns. The source was operated in the positive mode. An acceleration voltage of 25.0 kV (IS1) was applied for a final acceleration of 21.95 kV (IS2) using α-cyano-4-hydroxycinnamic acid (CHCA) matrix.

### 3.3. Preparation and Characterization of Alginate Nanoparticles and Loading of the Active Fraction E20

The alginate nanoparticles (Alg NPs) were obtained using a top-down process with Retsch ball mill (PM100S10, Eragny, France). For this purpose, 2 g of sodium alginate powder (Sigma Aldrich, St. Louis, MA, USA) and 112 g of zirconium oxide beads were placed in a grinding pot of 1.050 kg, for 10 h with a stirring at 440 rpm, and a pause every 5 min, followed by a reverse rotation every 10 min. The Alg NPs were collected and dispersed in Milli-Q water (500 µg/mL) under ultrasonication (Branson 2800, Branson Ultrasonics™, Brookfield, CT, USA) at a frequency of 40 kHz for 1 h at 25 °C. The E20 fraction was mixed at a concentration of 60 μg/mL with this suspension, leading to 12 wt.% of the Alg NPs’ concentration. This mixture was adjusted to pH 5 by addition of acetic acid (Sigma Aldrich). Size distribution (mean diameter and polydispersity index) and electrophoretic mobility of the nanoparticle dispersions were measured using dynamic light scattering (DLS) with a Malvern Zetasizer Nano ZS (Malvern Instruments Ltd, Worcestershire, UK). The measurements were made at 25 °C at a fixed angle of 173°. The size indicated was the average diameter of the nanoparticles (nm). The diameter was calculated from the correlation function of the intensity of the light scattered by the nanoparticles, assimilated to equivalent spheres. The polydispersity index (PdI) corresponded to the measurement of the homogeneity of the dispersion. Its value was comprised between 0 and 1. The zeta potential represented the surface charge of nanoparticles dispersed in a solution. It reflected the electrophoretic mobility of a charged suspension in an electric field. It was measured using the electrophoretic mode of the Zetasizer.

A solution of alginate nanoparticles (500 μg/mL) was prepared by ultrasonication for 30 min at 25 °C. A 1-mL sample was placed in the analysis cell. The following parameters were used: Analysis temperature = 25 °C; solvent = water; acquisition time = 5 s. For each sample analyzed, particle diameter, autocorrelation profile, size dispersion histogram (intensity versus diameter), percent polydispersity (% Pd), surface charge, conductivity, and deflection of zeta were verified. The measurements were made in triplicate.

### 3.4. High-Performance Liquid Chromatography (HPLC)

The yield of the E20 fraction adsorbed on the surface of the alginate nanoparticles was determined by HPLC. To this end, a solution of the E20 fraction loaded onto alginate nanoparticles was previously dialyzed on an 8-kDa cellulose membrane in order to remove the non-associated E20 compound with the nanoparticles. The sample was filtered through a 0.45-µm regenerated cellulose membrane before loading the column (injected volume, 40 μL). The equipment used during this experiment was the Shimadzu LC2010-HT (Shimadzu, Tokyo, Japan) with a column C4 of 5 μm QS Uptisphere^®^ 300 Å, 250 mm × 4.6 mm (Interchim, Montluçon, France). The eluent composition was a mixture of two solvents A and B (A: 0.1% trifluoroacetic acid in deionized water, and B: 0.1% trifluoroacetic acid in acetonitrile) with a flow rate of 1 mL/min. The column was heated to 40 °C, and the elution of the compounds was carried out by a linear gradient of 0 to 80% across 30 min. Sample detection was performed at 215 nm. The concentration of E20 was determined using the quantification of the obtained peaks area comparatively to samples of E20 with known concentrations analyzed under the same conditions. 

### 3.5. Scanning Electron Microscopy (SEM)

The SEM analysis of alginate nanoparticles was performed using an ULTRA 55 electron microscope (Zeiss, München, Germany), equipped with a thermal field emission transmitter and three different detectors (EsB detector with filtering grid, high-efficiency integrated lens SE detector, Everhart–Thornley secondary electronic detector). The parameters of the microscope were fixed as follows: Electron acceleration voltage: 200 V–20 KV; working distance: 1 mm; sub-nanometric resolution at 15 kV; beam current up to 100 nA. Several drops of the aqueous solution of alginate nanoparticles (500 μg/mL) were deposited on a silicon substrate previously cleaned in successive baths of acetone and isopropanol under ultrasonication across 10 min, and rinsed thoroughly with ultrapure water. Then the substrate was dried at 100 °C for 1 h before observation.

### 3.6. Minimal Inhibitory Concentration (MIC)

The minimal inhibitory concentration (MIC) is defined as the lowest concentration of a moleculethat inhibits the visible growth of a microorganism after overnight incubation in adequate conditions. MIC values of colistin (Sigma Aldrich, St. Louis, MO, USA), E20, and E20 loaded on alginate nanoparticles (Alg NPs/E20) were determined as previously described [24,44] with slight modifications. The E20 samples were diluted two-fold in sterile water adjusted to pH 5, as E20 was active only at this pH value. Then the obtained dilutions were incorporated in wells of a 96-well plate, and complemented with inoculated BHI broth with target strains at 10^7^ CFU/mL to a final volume of 250 µL. The 96-well plate was then incubated overnight at 37 °C and bacterial growth was checked visually. 

### 3.7. Time-Killing Curves 

The time-killing curves permitted us to establish the survival kinetics of *E. coli* 184 (*mcr*-1) and *E. coli* ATCC8739 in the presence of the E20 fraction and Alg NPs/E20 formulation. These strains at about 5 × 10^7^ CFU/mL, which were respectively resistant and sensitive to colistin, were inoculated in BHI medium containing colistin, the E20 fraction, or Alg NPs/E20 at their previously calculated MIC. As the activity of E20 was pH dependent, the samples and the control medium were adjusted to pH 5 with 1 M HCl, and were then incubated at 37 °C for 8 h. At regular intervals of time, a sample was taken out to determine the number of colony-forming units per mL (CFU/mL) in each condition tested by serial dilution and the colony counting method, after culture on BHI agar medium of each strain for 24 h at 37 °C.

### 3.8. Transmission Electron Microscopy (TEM)

The effects of alginate nanoparticles, E20, and Alg NPs/E20 on the cell morphology and the ultrastructure of the bacterial cells were assessed using TEM. The *E. coli* 184 strain, used as target bacteria, was treated with PBS (negative control), Alg NPs (500 μg/mL), E20 (1 mg/mL), or Alg NPs/E20 (500/60 µg/mL) during 18 h at pH 5, fixed using 2.5 % (*v*/*v*) glutaraldehyde solution and 0.1 M (*v*/*v*) cacodylate buffer (pH 7.4) and then placed on a Formvar film of 300 square mesh, nickel grid (EMS FF300-Ni). The TEM images were recorded on a JEOL JEM 2100FX TEM instrument at an acceleration voltage of 200 KV equipped with a GATAN CCD Orius 1000 camera and a GATAN CCD Orius 200D camera.

### 3.9. Proteins Quantification

The intracellular proteins from *E. coli* were quantified with a QuantiPro ™ BCA Assay Kit (Sigma Aldrich) upon treatment of the target bacteria with E20 fraction or Alg NPs/E20. To this end, 1 mL of fresh overnight culture of *E. coli* 184 grown in BHI medium was centrifuged (9000× *g*, 4 °C, 10 min). The resulting supernatant was eliminated, whereas the pellet was washed and resuspended in sterile phosphate buffer saline (PBS, pH 7). After centrifugation (9000× *g*, 4 °C, 10 min), the resulting pellet was treated with 1 mL of the following solutions: PBS (negative control), Alg NPs (500 μg/mL), E20 (1 mg/mL), or Alg NPs/E20 (500/60 µg/mL) at pH 5. Each treated pellet was incubated for 1, 3, and 5 h. At the end of the incubation times, each sample was centrifuged as above-cited in order to recover the supernatant. A standard protein solution was prepared using bovine serum albumin (BSA) at different concentrations in accordance with the manufacturer’s recommendations. After which, the protein concentration was determined in each sample (samples were diluted if necessary) after mixing with QuantiPro reagents and incubation for 1 h at 60 °C. After incubation, the optical density of the samples was measured using a spectrophotometer at 562 nm. The concentrations of extracellular proteins were then determined according the standard protein concentration curve. 

### 3.10. Cytotoxicity Assay

Two cell lines were used for cytotoxicity tests, IPEC-1 (Sigma Aldrich), an intestinal epithelial cell line derived from the small intestine of a newborn New Hampshire mini-pig, and HT29 human colorectal adenocarcinoma cell line (Sigma Aldrich). Cells were cultivated in 96-well tissue culture plates for seven days at 37 °C and 5% of CO_2_, in DMEM (Dulbecco’s modified eagle’s minimum) medium, supplemented with 10% fetal bovine serum, 5 mM of L-glutamine, and 100 U/mL each of penicillin and streptomycin (Invitrogen, France). The samples, E20 fraction, or Alg NPs/E20 at MIC values, prepared in DMEM medium, adjusted at pH 5 with acetate buffer or at pH 7, were added on the cells’ culture monolayer in wells and incubated for 24 h at 37 °C with 5% CO_2_. The acetate buffer alone and PBS control adjusted at the same pH values were also tested. After incubation, supernatants containing the samples were removed and the cell monolayer was washed with DMEM medium. CCK-8 assay (Dojindo Molecular Technologies, Japan) based on the reduction of tetrazolium salt by active mitochondria was used to assess cell viability of the treated cells. Then 200 µL of DMEM containing 10 µL of CCK-8 reagent was added in each well and cells were incubated for 2 h. Plates were then read at 450 nm in a microplate reader spectrophotometer (Xenius, Safas, Monaco). Results were expressed in % of absorbance at 450 nm observed with non-treated cells.

## 4. Conclusions

*Lacticaseibacillus paracasei* CNCM I-5369 produces antagonistic substances which were isolated as a mixture: The E20 fraction. Interestingly and encouragingly, this E20 fraction exhibited activity against different *E. coli* strains, including a strain carrying the *mcr*-1 gene. Here, we demonstrated that this anti-*E. coli* activity could be significantly enhanced when the E20 fraction was loaded on alginate nanoparticles (Alg NPs/E20). Besides this enhancement of activity, the nano-antibiotic formulation based on Alg NPs/E20 was devoid of cytotoxicity towards human HT29 and animal IPEC-1 cell lines. In this work, we reported an original finding which was the nano-antibiotic formulation based on E20 loaded on alginate nanoparticles. This approach represented an innovative and promising strategy to test in animal models before its application as a therapeutic option in fighting infections caused by GNB. 

## Figures and Tables

**Table 1:** Size and surface charge of alginate nanoparticles (Alg NPs) before and after loading of the E20 fraction.

Alginate Nanoparticles	Size by DLS (nm)	Zeta Potential (ζ) (mV)
Alg NPs (500 µg/mL) (pH 7)	111	−32
Alg NPs (500 µg/mL) (pH 5)	119	−12
Alg NPs + E20 fraction (pH 5)	124	0

**Table 2:** Inhibition diameter of Alg NPs/E20 at different pH values against *Escherichia* *coli* 184.

pH Value	Alg NPs/E20 (500/60) µg/mL
4	1–3 mm
5	3 to 6 mm
6	0–1 mm
7	0–1 mm

**Table 3:** Antibacterial activity of colistin, E20 fraction, and Alg NPs/E20 against different *E. coli* strains.

Strains	Colistin (MIC µg/mL)	Fraction E20 (MIC µg/mL)	Alginate Nanoparticles (500 µg/mL) + E20 (MIC µg/mL)
*E. coli* 184	8	1000	4
*E. coli* 289	8	2000	4
*E. coli* ATCC8739	2	1000	2
*E. coli* SBS36	2	1000	2
*E. coli* TOP10	1	250	2
*E. coli* E4A4	2	1000	2
*E. coli* E5A16	2	1000	2

MIC: Minimal inhibitory concentration expressed in µg/mL. The MIC is defined as the lowest concentration of an antibiotic that inhibits the visible growth of a microorganism after overnight incubation in adequate conditions.

**Table 4:** Bacteria strains used in this work.

Strains	Source/Origin
*Lacticaseibacillus paracasei* CNCM I-5369	Traditional Algerian cheese
*E. coli* 184 *	Al Atya et al. [25]
*E. coli* 289 *	Al Atya et al. [25]
*E. coli* ATCC8739	American Type Cell Collection, Manassas, VI (USA)
*E. coli* CIP 7624	Collection de l’Institut Pasteur, Paris (France)
*E. coli* SBS363	CEA, Saclay (France)
*E. coli* E4A4	ANSES, Ploufragan (France), from healthy pig feces
*E. coli* E5A16	ANSES, Ploufragan (France), from healthy pig feces
*E. coli* Top 10	Invitrogen, Carlsbad, CA (USA)

* Colistin resistant.
